# Supplementary material for: Modeling glioblastoma heterogeneity as a dynamic network of cell states
Source: Mol Syst Biol. 2021 Sep 16;17(9):e10105. doi: 10.15252/msb.202010105 (PMC8444284; doi:10.15252/msb.202010105)
Supplement: Supplementary file 6 — Source Data for Figure 5 [file MSB-17-e10105-s004.zip › Figure5A_sourcedata/GSEA_3017/nosigdb_stateE.GseaPreranked.1622795031093/SUVA_AC_PMID_31327527.html]

Details for gene set SUVA\_AC\_PMID\_31327527[GSEA]

|  || Dataset | state63017 |
| Phenotype | NoPhenotypeAvailable |
| Upregulated in class | na\_pos |
| GeneSet | SUVA\_AC\_PMID\_31327527 |
| Enrichment Score (ES) | 0.76919156 |
| Normalized Enrichment Score (NES) | 3.2719183 |
| Nominal p-value | 0.0 |
| FDR q-value | 0.0 |
| FWER p-Value | 0.0 |
Table: GSEA Results Summary

  

Fig 1: Enrichment plot: SUVA\_AC\_PMID\_31327527      
 Profile of the Running ES Score & Positions of GeneSet Members on the Rank Ordered List

  

| PROBE | GENE SYMBOL | GENE\_TITLE | RANK IN GENE LIST | RANK METRIC SCORE | RUNNING ES | CORE ENRICHMENT || 1 | PMP22 |  |  | 0 | 1.393 | 0.1452 | Yes |
| 2 | GFAP |  |  | 1 | 1.383 | 0.2893 | Yes |
| 3 | SPARCL1 |  |  | 7 | 0.963 | 0.3843 | Yes |
| 4 | FABP7 |  |  | 11 | 0.920 | 0.4769 | Yes |
| 5 | TTYH1 |  |  | 19 | 0.731 | 0.5456 | Yes |
| 6 | RAB31 |  |  | 35 | 0.598 | 0.5918 | Yes |
| 7 | F3 |  |  | 55 | 0.506 | 0.6242 | Yes |
| 8 | SLC1A3 |  |  | 66 | 0.472 | 0.6626 | Yes |
| 9 | CST3 |  |  | 86 | 0.425 | 0.6864 | Yes |
| 10 | GPM6B |  |  | 105 | 0.391 | 0.7078 | Yes |
| 11 | CLU |  |  | 119 | 0.364 | 0.7318 | Yes |
| 12 | GATM |  |  | 149 | 0.335 | 0.7356 | Yes |
| 13 | HEPN1 |  |  | 151 | 0.333 | 0.7692 | Yes |
| 14 | ATP1B2 |  |  | 260 | 0.258 | 0.6802 | No |
| 15 | DBI |  |  | 776 | -0.526 | 0.1824 | No |
Table: GSEA details [plain text format]

  

Fig 2: SUVA\_AC\_PMID\_31327527: Random ES distribution      
 Gene set null distribution of ES for **SUVA\_AC\_PMID\_31327527**
